# Exploring the Experience of Patients Who Received Mechanical Ventilation Support during Their Intensive Care Unit Stay

**DOI:** 10.3390/healthcare12141418

**Published:** 2024-07-16

**Authors:** Ruba Zeadnih, Imad Aljarrah, Ayman M. Al-Qaaneh, Maha Atout

**Affiliations:** 1 Faculty of Nursing, Al-Balqa Applied University (BAU), Al-Salt 19117, Jordan; ruba.zayadneh@bau.edu.jo; 2 Nursing School, Philadelphia University, P.O. Box 1, Amman 19392, Jordan; ijarrah@philadelphia.edu.jo; 3 Department of Allied Health Sciences, Al-Balqa Applied University (BAU), Al-Salt 19117, Jordan; ayman.alqennh@bau.edu.jo

**Keywords:** experiences, patients, mechanical ventilation, intensive care unit, Jordan

## Abstract

Objective: The objective of this study is to explore the experiences of patients who received mechanical ventilation (MV) support during their intensive care unit (ICU) stay in Jordan. Methods: A phenomenological hermeneutic approach was conducted, informed by the philosophy of Martin Heidegger. Fifteen patients participated in interviews regarding their experiences during their time in the critical care unit of three public hospitals in Jordan. Interpretive Phenomenological Analysis (IPA) was used to analyse the data. Findings: The findings of the current study indicated that patients who received MV support during their ICU stays experienced both physical and psychological suffering. A pattern of shared experiences among intensive care patients was identified. Five main themes captured the patients’ experiences: (1) feeling powerless, (2) being unable to recognise time, (3) feeling dead, (4) experiencing physical pain, and (5) having future concerns. Conclusion: The current study found that mechanically ventilated ICU patients suffer both physically and psychologically. Nurses should use specific strategies to relieve discomfort in addition to pain treatment. This is especially essential for non-verbal patients, whose actions may resemble those of their clinicians in pain.

## 1. Introduction

Intensive care, also known as critical care, is an organised, multidisciplinary, and paraprofessional system that provides intensive care and life support for acutely ill patients [1]. In the intensive care unit (ICU), we utilise technological devices and machines to support multi-organ system failure patients, particularly for the urinary, cardiovascular, and respiratory systems [2]. A breathing machine, also called a respirator or ventilator, is used in ICUs to help patients with respiratory problems that make breathing difficult [3].

Mechanical ventilation (MV) is a complex procedure in which positive breath pressure drives gas exchange, which is dependent on the compliance of the airway system. It is adopted when necessary to save and sustain the lives of critically ill patients in acute care settings [4]. Healthcare workers who care for acutely ill patients should have the necessary knowledge for the management of mechanically ventilated patients as well as be able to recognise its physiological and psychological effects on patient health status [3].

Numerous studies have shown that a substantial number of ICU patients require ventilator support at some point during their stay, with percentages ranging from 30% to 98% [5,6]. Furthermore, Whunsch et al. reported that 52% of patients younger than 65 years hospitalised in six states in the United States (US) received MV [7]. Moreover, a longitudinal study conducted to investigate the benefit of ventilation therapy among hospitalised patients diagnosed with COVID-19 utilising real-world data of more than half a million hospitalised adult patients older than 18 years revealed that 10% of the participants received ventilation therapy, while patients with a mean age of 52 years diagnosed with chronic respiratory diseases and malignancy benefitted from ventilation therapy compared with other cases [8].

Despite its lifesaving benefits, MV can also have a significant negative impact on patients’ experiences in the ICU. Many ICU survivors have described their experience while receiving MV as frightening and upsetting, with the most stressful event of this experience being the communication impairment that resulted from MV support [9,10]. Understanding the experiences of mechanically ventilated patients in the ICU is essential for healthcare providers to provide patient-centred care and improve patient outcomes.

A systematic review and meta-analysis conducted by Razban and his team revealed that one-third of ICU survivors had anxiety symptoms that persisted for 12 months after ICU discharge. Additionally, patients who were treated with MV were four times more likely to have delusional memories than non-ventilated patients [11]. Another phenomenological study conducted by Marasinghe et al. explored the experiences of 15 mechanically ventilated patients (8 females and 7 males) who, following admission to the ICUs of three hospitals in Sir Lanka, were given a semi-structured interview. These interviews, after being analysed using a thematic analysis, revealed that the participants had suffered from pain and sore throat due to endotracheal intubation, painful suctioning, and irritation, as well as feelings of thirst, back pain, fear, and anxiety, and were unable to express their feelings. Furthermore, poor communication and an inability to express their feelings increased patients’ stress and frustration [12].

Several studies have aimed to identify strategies that can mitigate the negative impact of being mechanically ventilated in ICUs. Azimi et al., in a cross-sectional study, found that spiritual health enhanced the ability of ICU patients who were mechanically ventilated to cope with emotionally arousing memories. Furthermore, quality communication between patients and healthcare providers in Iran has significantly mediated the development of these memories [13]. Another cross-sectional study conducted by Thapa, Dahal, and Singh (2019) to assess communication difficulty among 48 mechanically ventilated patients in ICUs indicated that the vast majority (98%) of these patients reported sleep disturbances and communication difficulties. Furthermore, the results of the study found that most of the patients used their hands for pointing and gesturing, more than 65% used shaking hands, and less than 20% used writing [14]. Accordingly, developing alternative communication methods and enhancing the awareness of healthcare providers regarding communication difficulties will reduce the psychological stressors caused by ineffective communication.

In a qualitative study that aimed to describe the experience of eight mechanically ventilated patients in an ICU in the northern part of Sweden via personal interviews, Engestrom et al. [15] found that the analysis resulted in two themes and seven categories: (i) being delivered into the hands of others (feeling vulnerable and dependent, struggling to be able to communicate, feeling safe with the staff, and being cared for in an unknown environment) and (ii) the unlikely perception of the reality (relatives were there and were taken care of, memories and perception of time varied and appreciating the daily and follow-up visits) [15]. Another qualitative study was conducted by Glider et al. using inductive thematic analysis for 10 post-cardiac surgery patients who were recruited to explore the experience of the endotracheal tube (ETT) after MV revealed three themes: (i) the experience of the ETT and intubation, (ii) the experience of emerging from sedation, and (iii) participants’ concerns about the future. The patients described the presence of the ETT as ‘bothersome’ and breathing through it and the extubation process as ‘weird’ and ‘strange’ [16].

Accordingly, understanding the experiences of mechanically ventilated patients in the ICU is important, as it can provide valuable insights into the quality of care provided in the ICU. It can also help in identifying areas for improvement and assist healthcare providers in delivering patient-centred care. By exploring patients’ experiences, it is possible to gain a better understanding of the psychological and emotional challenges and long-term outcomes and prognoses faced by mechanically ventilated patients, all of which can collectively enhance the development of interventions and support strategies that address the holistic needs of these patients.

Unfortunately, no studies have been conducted in Jordan regarding the experiences of mechanically ventilated patients in the ICU. Accordingly, the objective of this study was to explore the experiences of patients who received MV support during their ICU stay in Jordan.

## 2. Methodology

The aim of this study was to explore the experiences of patients who received MV support during an ICU stay. A hermeneutic phenomenological approach was conducted, informed by the philosophy of Martin Heidegger. The study was conducted in ICU units in several hospitals in Jordan (Prince Hamza, Al-Basheer, and Jerash).

### 2.1. Sampling and Recruitment

Patients were recruited from three public hospitals in Jordan. After receiving ethical approval from Balqa University to conduct the study (2024\2023\1\6), the researcher met with the nursing administration of the ICU units to discuss the aims and objectives of the study. The head nurses of the recruited units were provided with the inclusion and exclusion criteria to find eligible participants within their units. A purposeful sampling strategy was adopted to recruit the participants. The inclusion criteria for patients were chosen for interviews (see Table 1).

Invitation letters that included information sheets were given to all participants who were eligible to participate in the study. Patients who agreed to participate in the study were contacted by the researcher to discuss the study and sign the consent forms, as well as to determine the time of the interview.

### 2.2. Data Collection

Individual open interviews were held with adult patients who had received MV support. To portray multiple views of the phenomenon of interest, several semi-structured interviews were conducted with parents to explore their experiences of receiving MV support. Open-ended, semi-structured interviews were recommended because they allow the discussion to flow and show the complexities of the phenomena, which are unique to certain contexts or give rise to a pattern across the contexts [17]. Initially, interview questions were developed based on the literature review; however, they were modified after the transcription and primary analysis of the interviews when new insights and ideas emerged (see Table 2).

### 2.3. Data Analysis

The present study utilised an integrative phenomenological analysis technique [18]. The duration of each interview varied from 15 to 30 min. The analysis was carried out in the following manner: The main researcher listened carefully to the interviews and transcribed them word for word. The manuscripts were meticulously scrutinised, and each line was examined repeatedly. Initial descriptive, linguistic, and conceptual observations were recorded in the margin of each transcript, and the transcripts were entered into Nvivo 12. Subsequently, the texts were reviewed once more, and the data were organised to detect any modifications in the data and identify emerging patterns. The primary researcher attempted to decrease the amount of specific information in order to document the patterns and connections among the notes. The initial codes were abstracted and then organised into clusters based on shared characteristics in terms of meaning. Upon completing a transcript, the researcher conducted a re-analysis of the themes within the context of the dialogue to verify the accuracy and appropriateness of the meanings for the participants. This procedure was iterated until all the transcripts had been scrutinised. Ultimately, the researcher identified the patterns and themes that encompassed all instances expressing the various meanings conveyed by the texts.

### 2.4. Ethical Considerations

The Human Research Ethical Committee (IRB) received ethical approval to conduct the study. The study participants were informed about the objectives of the study and the voluntary nature of their participation. The participants were provided with an information sheet regarding the study. The participants were provided with a 72 h timeframe to decide on their participation in the study and were notified that they had the option to withdraw from the study at any point. A consent form was acquired in writing. The participants’ confidentiality was ensured by anonymising their names and any other identifying information, such as using numerical identifiers.

### 2.5. Trustworthiness

The study utilised Guba and Lincoln’s paradigm to assess trustworthiness [19]. Each participant was provided with a summary of the interview to verify the accuracy of the descriptions. The interviewer confirmed the primary findings with the participants. Two investigators independently evaluated all transcripts and individually created themes. Subsequently, these ideas were discussed, resulting in the identification of a shared understanding. The researchers possessed expertise in providing care in ICUs. In addition, the ward nurse was consulted regarding each patient’s physical and cognitive capacity to take part in the study. If there was any indication of ambiguity, the patient was automatically excluded.

## 3. Findings

### 3.1. Patient Characteristics

The sample consisted of 15 patients. Table 3 displays a description of the participants. Roughly, more than 50% of the patients were admitted as a result of cardiac illnesses, while the remaining patients had respiratory and kidney illnesses.

### 3.2. Themes

Five major themes reflect the experiences of patients who received MV support during their ICU stays in Jordan. The themes include feeling powerless, unable to recognise time, feeling dead, physical pain, and concern for the future.

#### 3.2.1. Feeling Powerless

The majority of patients (1, 2, 6, 7, 8, 12, 13, and 15) reported having feelings of powerlessness when they found themselves on the mechanical ventilator. This feeling came from being unable to communicate with their families and healthcare providers:

‘I felt that my body was mummified and unable to move… I heard you guys talking about Friday prayers, but I felt like I was embalmed, and I couldn’t react.’

(Patient 5)

A few patients (1, 4, 5, and 8) mentioned that nobody heard them while they were on the ventilator. This caused them to feel sad, as some of them felt that they were abandoned and ignored:

I was trying to call my brother, who didn’t hear my voice, and suddenly, he stood up and walked away from me and left me. I thought he had abandoned me, and I was very sad… When I got healthy and asked him whether he intentionally left me, he told me, ‘When you were opening your eyes and trying to move, the nurses told me to go and inform them to give you a sleeping medicine’, so that he was calling the nurses to give me medicines.

(Patient 1)

Patient 2 mentioned that she was unable to raise her hand when the doctor asked her to do so. Although she mentioned that she heard the doctor very well, she was unable to raise her hand:

I was listening to them. The doctor came and began to talk with me… ‘Open your eyes. Hajjah, raise your hand’. I’m listening to them, but I’m not able to respond to them. I was myself telling my son to raise my right hand or raise my leg as much as I could. The doctor opened my eyelids with his hand.

(Patient 2)

Several patients mentioned that they felt tied to several tubes, which caused them to feel irritable and disturbed. Moreover, some patients felt upset, as they felt that their hands were tied to prevent them from removing the tubes:

I tried to remove it, but I couldn’t, because I was stuck. Then the nurses came and tried to calm me down and told me to please cooperate with us in order for us to remove the device and for me to return to breathing.

(Patient 3)

#### 3.2.2. Unable to Recognise Time

Most of the patients mentioned that they had lost their ability to distinguish time when they were on a ventilator machine. They mentioned that this increased their anxiety:

I did not feel in time. This means that I did not know in what time we were now… and this increased my anxiety, I was still working… and the time was very important to me and suddenly I lost my feeling of time… I felt that I was disconnected from the world. This was really difficult.

(Patient 3)

Patient 2 felt annoyed that he was unable to distinguish the night from the morning:

And the most annoying thing was not knowing the time. I have to know the time—everything that my wife misses. I asked her, ‘Are we day or night? Especially at the stage when they seemed to remove the device when they woke me up, and the tube was still in my mouth. This stage was very difficult… I had to ask them about the time because I couldn’t distinguish the night from the morning.

(Patient 2)

Patient 1 wanted to know the time to pray:

I can remember when they were trying to remove the tube from my pharynx… they were trying to awake me gradually. I was unable to know the time and what the time to pray was. My husband came and told me, ‘Sumaya. Pray even in your eyes, or pray two prayers together.’

(Patient 1)

Other patients (7, 8, 11, and 14) mentioned that the ICU department was full of light, so they were unable to tell the time, whether it was day or night:

I did not know the time, as the room I was in had no windows, and the light was on 24 h, even for the nurses. I asked them, ‘Do you work 24 h a day? I kept seeing him in front of me, but then I would sleep for five minutes, but I thought that I slept for five or eight hours, so I did not know how long I slept. This is why I told them that they worked 24 h.

(Patient 11)

Patient 14 mentioned that the time was very long, and he was unable to do anything during this long time:

When I was sleeping, I felt that it was three to four hours… But when I asked about the time, they told me five minutes more than the last time I asked. This means that the day was very long… Of course, I do not know what I did during the six hours.

(Patient 14)

#### 3.2.3. Feeling Dead

Several patients (1, 2, 4, and 8) were unable to distinguish whether they were alive or had died. They thought that they were in the death stage:

The most I suffered was when I heard the crying of the people around me. I thought that I had died.

(Patient 1)

When I arrived at the hospital, I heard people around me crying hysterically. I thought that they were having my funeral because I had died, and this was the funeral home.

(Patient 8)

Patient 4 was scared of sleeping, as he thought that he might die at any moment:

I did not know what to say… my daughter… but it is a horrible feeling, a scary feeling. I was afraid to close my eyes. Even until now I am scared if they do not wake me, and I am scared that I will die.

(Patient 4)

I was thinking of death–only death. I had nothing on my mind except death. The speech was lost, and the question was lost… everything was lost. I wanted to speak, but I was unable to. I felt myself as being separate from the world.

(Patient 2)

#### 3.2.4. Physical Pain

Several patients (8, 10, 11, 12, 13, 14, and 15) mentioned the physical pain and disturbances that they felt because of the endotracheal tube being inserted or removed from their pharynx:

‘Pain… pain… I was tired, and when they removed it… they removed my soul with it.’

(Patient 10)

Moreover, Patient 10 mentioned that the suction process was very painful for him:

When they awakened me… when they wanted to remove the tubes and before that when they inserted it to suction the fluid from the operation; this removed the soul… very painful, very painful. They told me, ‘We want to remove the tube and then insert it again’. This is similar to Sikh.

(Patient 10)

Patients 11 and 15 mentioned that the most difficult experience involved the endotracheal tube. Patient 11 felt relieved directly after it was removed:

‘The most difficult experience is the air tube, and they do not remove it directly after you awake. They wait two to three hours.’

(Patient 11)

Patient 12 mentioned that the healthcare team tried to insert the endotracheal tube more than once, and this increased his pain:

‘When they inserted the tube… They tried two to three times. This was accompanied by pain.’

(Patient 12)

Patients 13, 14, and 15 felt that the tube closed their mouths and throats, and each was unable to speak:

‘I felt that it closed my mouth and throat and prevented me from speaking.’

(Patient 13)

#### 3.2.5. Concerns about the Future

Several patients (1, 4, 5, 8, and 11) described their concerns while they received MV support during their ICU stay. These concerns arose from their fear of death and leaving their families and loved ones, especially those in need of them:

I have fears about my children. I have one son who prepares for a master’s degree in math and two daughters who have Tawjihi [last school year]. Before having the operation, I continuously coordinated things in case I did not survive… Their lives had to be continued, and they had to live in a normal way. This meant that I had the feeling that at any moment things might completely change… This meant a change from positive to negative. So, if things changed suddenly, how would they live after me? So I acted in a way that enabled them to live well after me.

(Patient 5)

## 4. Discussion

The aim of this study was to investigate the living experiences of patients who underwent mechanical breathing assistance throughout their stay in the ICU in Jordan. The findings of the current study demonstrated that the majority of patients reported a feeling of powerlessness when they found themselves on MV. The sensation of powerlessness might be related to the absence of authority over their own physical beings and the dependence on medical technology to sustain their lives. Patients may experience a sense of confinement, powerlessness, and vulnerability in relation to their healthcare providers. This feeling of powerlessness can result in heightened levels of anxiety, despair, and the sensation of being socially disconnected. This finding is congruent with several studies conducted in the literature, for example, Hajiabadi et al. [20].

The current study found that several patients reported having difficulties communicating with nurses and families as a result of being connected to the mechanical ventilator. This sense of confinement and powerlessness can lead to increased feelings of anxiety, fear, and frustration for patients. This is congruent with several studies [21,22]. Kyranou, Cheta and Pampoulou [21] investigated the challenges that nurses in Cyprus encountered while attempting to communicate with aware patients undergoing mechanical breathing. The absence of sufficient training for nurses and the lack of suitable technology for augmentative and alternative communication have resulted in the neglect of the intricate communication requirements of critically ill patients.

A few patients mentioned that they had lost their ability to distinguish time when they were on a ventilator machine. At that time, patients experienced disorientation and a sense of detachment from reality due to their lack of time awareness. This finding is congruent with a study conducted by Karlsson, Bergbom, and Forsberg [23]. This study found that patients who were conscious during MV in an intensive care unit lost track of time and were oblivious to the distinction between day and night, as well as uncertain about when they would be free from the tube.

Several patients were unable to distinguish whether they were alive or had died. They thought themselves in the death stage. Their confusion and disorientation exacerbated their fear and discomfort, making it even more difficult for them to comprehend their present circumstances. These patients found themselves in a situation of permanent uncertainty as the distinction between life and death became blurred.

Furthermore, several patients mentioned the physical pain and disturbances they felt because of the ventilator being inserted or removed from their pharynx. This is congruent with several studies reported in the literature [16,20,22]. For example, a study conducted by Gilder et al. [16] found that the presence of the endotracheal tube was described as bothersome, while breathing through the tube and extubating were described as ‘weird’ and ‘strange’ but not painful. However, the patients in the current study mentioned that they felt pain when the endotracheal tube was removed.

In the current study, patients mentioned that they were afraid in terms of their families and how they might die and leave them. They had significant fears of leaving their children without anybody to help them in life. This is congruent with the findings of several studies [16,20] that participants expressed apprehensions regarding the future, such as their desire to witness the growth of their family and make lifestyle modifications that would have a beneficial effect on their future well-being.

The current study’s findings have important implications for improving the experiences of patients who receive MV support during their ICU stays, for example, the early administration of analgesics for comfort while minimising any pain. However, evaluating pain in the ICU setting has challenges as patients frequently lack the ability to express themselves verbally due to factors such as the severity of their illness, the use of an endotracheal tube, and the use of sedating drugs. Therefore, several objective pain assessment tools could be used to evaluate pain in non-verbal adult patients in intensive care units (ICUs). The measurements have been classified as either one-dimensional, which uses behavioural scales, or multidimensional, which are objective measures that assess two or more pain dimensions, including behavioural and physiological responses [24].

Moreover, it is important to promote sleep, active involvement of the family, early mobilisation, and consistent communication from the time of admission to the end of the ICU stay. This approach encompasses not just pain relief and relaxation but also the personalised control of various aspects of discomfort. Moreover, it is important to use personalised approaches when giving comfort rather than relying solely on universal pharmaceutical therapies.

Implementing strategies such as the use of communication aid supplies by mechanically ventilated conscious patients can facilitate communication and subsequently reduce the anxiety levels in these patients [25]. For example, the utilisation of communication boards in conscious patients who are mechanically ventilated resulted in an improved ease of communication. Moreover, actively engaging patients in the decision-making process and offering emotional support can effectively empower patients and enhance their overall well-being during this difficult period [22].

Therefore, it is imperative to provide training to nurses and families on the appropriate methods of communication with patients during their stay in the ICU, such as giving priority to conducting regular evaluations of communication obstacles and swiftly implementing treatments to address them. This research has the potential to provide valuable insights for undergraduate nursing programs, enabling them to better prepare future professionals in effectively communicating with aware patients who are on mechanical ventilation. By tackling these obstacles, we can improve the overall standard of care and increase the patient experience of individuals undergoing MV.

The current study has several limitations. First, due to the limited sample size, it is difficult to make broad generalisations from the findings of this study. However, employing a qualitative methodology prioritises gaining a comprehensive understanding of the individuals’ profound experiences rather than drawing generalised conclusions from the findings. Therefore, the findings can be applied to contexts that share similar characteristics. Also, as patients included in the study were selected by the nursing director, this may possess a selection bias. In addition to that, the study depends on the ability of the patient to recall his/her experience while on MV, which may be limited for some patients. Furthermore, the current study was conducted with only patients. Therefore, it is imperative to conduct another study to explore the viewpoint of healthcare providers on this phenomenon.

## 5. Conclusions

Although this is the first study that assesses the perceptions of patients on MV in Jordan, the results of the current study are consistent with other studies worldwide. The findings of the current study indicate that patients who received MV support during their ICU stays experienced both physical and psychological suffering. Accordingly, nurses should take into account these two elements that contribute to patients’ discomfort. Nurses should not focus solely on pain management but also utilise other targeted methods to alleviate discomfort. This is particularly important for non-verbal patients, whose behaviour may resemble that of their clinicians when they are in pain. Researchers recommend that university nursing education programs and hospitals’ professional development programs should include the perceptions of MV patients in their curricula. Further research is required to assess the change in patients’ perceptions in the presence of other medications or other contributing factors.

## Figures and Tables

**Table 1:** Inclusion and exclusion criteria.

**Inclusion Criteria**
Treatment with invasive mechanical ventilation Ability to read, write, and provide their own written informed consent Glasgow coma scale 15
**Exclusion Criteria**
Patients with cognitive impairment Patients diagnosed with any psychiatric illnesses

**Table 2:** Interview guide questions.

Question
Please tell the story of your illness since the beginning of your diagnosis with this disease. Tell me about your stay in the ICU. Do you remember any special events or experiences? Describe in detail. What difficulties/suffering did you face during your stay in the intensive care unit? How did you feel when you were put on a ventilator when you entered the intensive care unit? Tell me more about the details of this experience.

**Table 3:** Participants’ characteristics.

Participant (Interview #)	Age (Years)	Gender	Admission Diagnosis	Type of Intubation	Type of ICU	ICU LOS (Days)	Post-Extubation Day of Interview	Days on Mechanical Ventilation
1	48	Female	Respiratory failure	Non-elective	General	14	10	4
2	70	Female	Acute MI	Non-elective	General	4	2	2
3	63	Male	Acute kidney injury	Non-elective	Medical	4	1	2
4	60	Male	ACS	Elective	Cardiac surgical	7	6	1
5	63	Male	MI	Elective	Cardiac surgical	19	9	2
6	69	Male	ACS	Non-elective	Cardiac surgical	7	3	1
7	59	Male	ACS	Elective	Cardiac surgical	20	9	2
8	62	Female	Pulmonary edema	Nonelective	Medical	3	1	1
9	59	Male	ACS	Elective	Cardiac surgical	12	1	1
10	46	Male	ACS	Elective	Cardiac surgical	11	1	1
11	64	Male	ACS	Elective	Medical	6	2	1
12	68	Male	COPD	Non-elective	Medical	3	1	1
13	83	Female	PO	Non-elective	Medical	4	1	1
14	54	Male	Aortic valve stenosis	Elective	Cardiac surgical	7	1	4
15	59	Male	MI	Elective	Cardiac surgical	7	1	2

#: number; ACS: Acute coronary syndrome; MI: Myocardial infarction; COPD: Chronic obstructive pulmonary disease; LOS: length of stay; PO: pulmonary oedema.

## Data Availability

Data are contained within the article.
